# Effectiveness of the Internet of Things for Improving Health of Non-Pregnant Women Living in High-Income Countries: A Systematic Review

**DOI:** 10.3390/healthcare13243310

**Published:** 2025-12-17

**Authors:** Olukunmi Omobolanle Balogun, Etsuko Nishimura, Noyuri Yamaji, Kiriko Sasayama, Md. Obaidur Rahman, Katharina da Silva Lopes, Citra Gabriella Mamahit, Mika Ninohei, Phyu Phyu Tun, Rina Shoki, Daichi Suzuki, Aya Nitamizu, Windy Mariane Virenia Wariki, Daisuke Yoneoka, Eiko Saito, Erika Ota

**Affiliations:** 1 Graduate School of Public Health, St. Luke’s International University, Tokyo 104-0045, Japan; 2 Faculty of Nursing, Komazawa Women’s University, Tokyo 206-8511, Japan; 3 Graduate School of Nursing Science, St. Luke’s International University, Tokyo 104-0044, Japan; 4 Institute of Clinical Epidemiology, Showa Medical University, Tokyo 142-8555, Japan; 5 Department of Family Nursing, Graduate School of Medicine, The University of Tokyo, Tokyo 113-8654, Japan; 6 Sustainable Society Design Center, Graduate School of Frontier Sciences, The University of Tokyo, Chiba 277-8561, Japan; 7 Department of Epidemiology, National Institute of Infectious Diseases, Japan Institute for Health Security, Tokyo 162-8640, Japan; 8 Center for Evidence-Based Medicine and Clinical Research, Dhaka 1212, Bangladesh; 9 Faculty of Health Science, University of Potsdam, 14469 Potsdam, Germany; 10 Nezu Biotech GmbH, 69121 Heidelberg, Germany; 11 School of Nursing, Dokkyo Medical University, Tochigi 321-0293, Japan; 12 Faculty of Nursing at Saitama, Japanese Red Cross College of Nursing, Saitama 338-0001, Japan; 13 Institute for Global Health Policy Research, Bureau of Global Health Cooperation, Japan Institute for Health Security, Tokyo 162-8655, Japan; 14 School of Nursing, Tokyo Medical University, Tokyo 160-8402, Japan; 15 Department of Community Medicine, Faculty of Medicine, Sam Ratulangi University, Manado 95115, Indonesia; 16 Graduate School of Medicine, The University of Tokyo, Tokyo 113-0033, Japan

**Keywords:** Internet of Things, IoT, women’s health, mHealth, digital health, health app, systematic review

## Abstract

**Background/Objectives**: There is increased advocacy for the potential for digital applications (apps) and the Internet of Things (IoT) to improve women’s health. We conducted a systematic review to assess and synthesise the role of Apps and the IoT in improving the health of non-pregnant women. **Methods**: Six databases were searched from inception to 13 February 2023. We included randomised controlled trials that assessed the effects of various Apps and the IoT with regard to improving the health of non-pregnant women in high-income countries. Our primary outcomes were health status and well-being or quality of life, and we assessed behaviour change as the secondary outcome. Screening, data extraction, and quality assessment were performed in duplicate. Study quality was assessed using the Cochrane Risk of Bias 2.0 tool. Narrative methods were used to synthesise study outcomes. **Results**: The search retrieved 18,433 publications and seven publications from six studies met the inclusion criteria. Participants included overweight or obese women, postmenopausal women, or women with stage I–III breast cancer. Intervention types varied across included studies but broadly included wearable or sensor-based personal health tracking digital technologies. The most commonly assessed intervention effect was on behaviour change outcomes related to promoting physical activity. Interventions administered yielded positive effects on health outcomes and well-being or quality of life in one study each, while three of the four studies that assessed behaviour change reported significant positive effects. Most included studies had methodological concerns, while study designs and methodologies lacked comparability. **Conclusions**: Based on our findings, the use of apps and the IoT may be promising for facilitating behaviour change to promote physical activity. However, more evidence is needed to assess the effectiveness of the IoT for improving health status, well-being and quality of life among non-pregnant women.

## 1. Introduction

Women’s health has historically had an exclusive focus on gynaecological and reproductive health, causing a neglect of women’s health needs beyond reproduction [1,2]. However, health priorities for women encompasses much more than reproductive health [3], especially in view of current demographic and epidemiologic transitions. It is well established that women live longer than men globally [4]. However, women are also reported to have more disability and problems with physical functioning during their lifetime compared with men [4,5]. For example, obesity prevalence is higher in women and is more strongly associated with hypertension, cancer, and depression than in men [6]. Women are also shown to have higher mortality and worse prognosis after acute cardiovascular events [7].

Despite the conventional viewpoint that women in high-income countries (HICs) tend to utilise more healthcare [8,9,10] and preventive care services [11], they also face systematic challenges wherein gender differences in disease management are often disadvantageous to women [12]. Hence, gender disparities in access to and quality of healthcare, often leads to delayed diagnosis [13,14] and higher burden of morbidity-driven conditions in women [15]. The ensuing inequality in healthcare has been widely reported [13,14,16] with the underdiagnosis and undertreatment of women attracting attention as a global public health concern [17]. In view of the foregoing, many organisations and societies are prioritising the health of women beyond reproductive health [17,18,19]. Furthermore, there is increased advocacy for the potential for digital health technologies to improve women’s health and promote equity [20].

The World Health Organisation (WHO) defines digital health as a field of knowledge and practice associated with the development and use of digital technologies to improve health [20]. It also encompasses other uses of digital technologies for health such as the Internet of Things (IoT). The IoT is a system of interrelated digital devices that are capable of data exchanges over a network without human-to-human or human-to-computer interactions [20,21,22]. Following the introduction of smart healthcare in 2009, attempts to use digital and other technologies to manage information related to people’s health and address healthcare needs have been expanded [23]. Among IoT interventions, smart healthcare systems are used for disease prevention and health improvement. Smart healthcare is mainly used in home care, self-care, and acute care settings, where self-care systems allow people to monitor their own health conditions and have access to information through wearable devices and smartphones [21]. Accordingly, previous studies have revealed the effectiveness of IoT in improving health outcomes [24,25,26] and encouraging lifestyle behavioural changes [25,26,27,28]. For example, a systematic review on the effectiveness of personalised mobile interventions on lifestyle behaviours within a mixed population (64% of which were women) reported improved lifestyle behaviours [28].

Recently, the IoT is increasingly being leveraged to promote and achieve improvement in women’s health globally. Studies also show that women who wish to participate more in their health issues often use IoT linked devices for day-to-day lifestyle monitoring [27] or management of chronic conditions [22,24]. Examples of application of IoT in women’s health include health monitoring during pregnancy and postpartum period [29], sensor-based menopause transition monitoring [30], interventions for reducing risk of postpartum weight retention [31], and assisted technologies to prevent sarcopenia [32]. Such applications of IoT, which can be used daily and continuously may help address some of the healthcare needs and challenges that women face. Additionally, the use of IoT-based applications may provide opportunities and encourage improvements in women’s health by enabling them to make informed choices and engage in healthy lifestyle behaviours. IoT use may also have the potential to reduce gender disparities in healthcare through improved access to required healthcare services.

Types of IoT interventions currently employed for women’s health vary widely, ranging from mobile phones, smart bands and wearable devices for tracking steps, exercise and sleep, to sensor-based devices, or a combination of these which can measure health data and connect to the internet [22,33]. A recent review on the use of IoT interventions during pregnancy and postpartum period suggested that IoT interventions may limit gestational weight gain in pregnant women with obesity [29]. Despite the growing application of IoT to women’s health, the effectiveness of such interventions among non-pregnant women has not been systematically assessed, and there is no true consensus about the effectiveness of the IoT in improving women’s health outcomes [28]. Therefore, a rigorous evaluation that considers all forms of the IoT was planned, for generating evidence and promoting appropriate integration and usage of technologies within existing health systems in order to improve women’s health outcomes [34].

## 2. Materials and Methods

This review was performed according to the protocol registered in the International Prospective Register of Systematic Reviews (PROSPERO CRD42022384620), and adhered to the Preferred Reporting Items for Systematic Reviews and Meta-Analyses (PRISMA) guidelines (Table S1) [35]. The protocol for this review was previously published [34].

### 2.1. Search Strategy

We performed an electronic search of PubMed (including MEDLINE), Cochrane Central Register of Controlled Trials, Embase, Cumulative Index to Nursing and Allied Health Literature (i.e., CINAHL), and PsycINFO to identify any relevant studies that met our eligibility criteria. We also searched ClinicalTrials.gov (clinicaltrials.gov) and the WHO International Clinical Trials Registry to identify additional ongoing studies. All databases were searched on 13 February 2023, from inception with no restrictions on language or publication dates. The search strategy was developed using keywords and controlled vocabulary related to participants (woman), intervention (IoT, e.g., mobile application, wearable electronic device, and smart device), and study design (randomised controlled trials). Consistency in the theme was applied by considering terminological and technical differences between the databases. Supplementary searches also included a backwards citation search of all included studies and systematic reviews. Two experienced librarians developed and executed the search strategy shown in Supplementary Materials S1.

### 2.2. Inclusion and Exclusion Criteria

The inclusion and exclusion criteria were developed using the PICOS criteria for population, intervention, comparison, outcome, and study design (Table 1). Randomised controlled trials (RCT) and cluster-RCT studies examining the role of applications (apps) and IoT in improving women’s health compared with the use of standard care, no intervention, or another intervention (e.g., education or exercise without IoT) were included. This review included only studies that examined intervention effect among non-pregnant working-aged women. We excluded studies with mixed population unless data was presented separately based on gender or those that included over 80% of female participants who met the inclusion criteria.

### 2.3. Study Selection

The results from the database searches were imported into EndNote X9 and de-duplicated. These records were then exported to Rayyan, an online screening tool [36] for title and abstract screening. Twelve review authors (E.N., N.Y., K.S., P.P.T., M.O.R., M.N., G.M., K.DS.L., R.S., D.S., A.N., H.H.) working independently in pairs conducted the initial title and abstract screening. Following the title and abstract screening, we retrieved full text of studies that were included by at least one reviewer. All stages of screening were conducted in duplicate amongst review authors, and reviewers were blind to each other’s decisions. Any discrepancies in screening decisions were resolved through discussion between the reviewers, or in consultation with a third reviewer when required.

### 2.4. Data Extraction

Six review author pairs (E.N., N.Y., K.S., P.P.T., M.O.R., M.N., G.M., K.DS.L., R.S., D.S., A.N., H.H.) independently extracted data from the included studies using a predesigned data extraction form in Microsoft Excel (Microsoft Inc., Redmond, WA, USA). Extracted data included relevant study information (study setting, country, research aims, participants, interventions, comparisons, study design, and outcome measures, including primary and secondary outcomes) and results pertaining to each eligible outcome. Discrepancies were resolved through discussion or by consulting with a third reviewer (E.O.). In case of any unclear or missing information, attempts were made to contact the authors to collect relevant data.

### 2.5. Risk of Bias

The quality of the individual studies was assessed using the Cochrane Collaboration’s risk of bias assessment tool 2.0 [37]. Five review author pairs (O.O.B., N.Y., K.S., P.P.T., R.S., D.S., A.N., E.N., M.O.R., W.M.V.W.) independently assessed the risk of bias for individual review outcomes on each of the following domains: those related to the randomisation process; deviations from intended interventions; missing outcome data; measurement of the outcomes; and selection of the reported results. Supporting text for the judgement of risk of bias domain was provided for each assessment. Risk of bias for each domain and the overall risk of bias for each study was classified into three categories: low risk of bias, some concerns, or high risk of bias. Any discrepancies were resolved through discussion or in consultation with a third reviewer.

### 2.6. Data Synthesis

A descriptive overview of the characteristics of the included studies is presented using narrative summaries and tables with key information. We planned to perform network meta-analysis separately on women’s age categories and medical history to estimate the direct, indirect and relative effect of IoT interventions on each outcome. However, the quantitative outcome data were deemed insufficient to pool in a network meta-analysis due to the few numbers of included studies [38,39]. Also, meta-analysis could not be performed due to heterogeneity in participant characteristics across studies [39]. Included studies assessed different outcome measures using varying assessment methods and introducing substantial inconsistency in the reported effect metrics across studies. Results were therefore summarised in tables and synthesised narratively.

## 3. Results

### 3.1. Search Results

**Figure 1:**
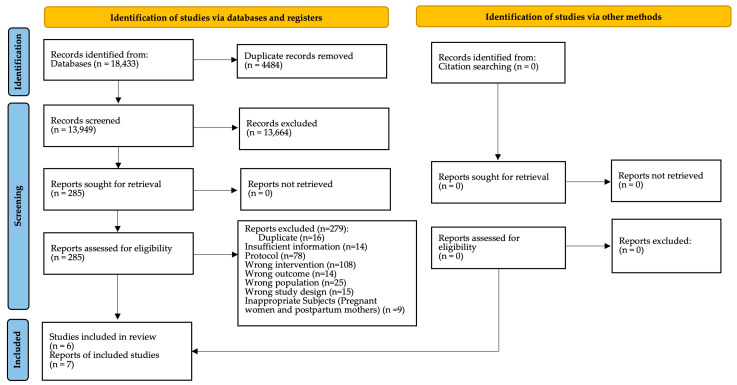
Flow diagram of search results and study selection.

The database searches yielded 18,433 records. After duplicates were removed, a total of 13,949 records remained for title and abstract screening. Full texts of 285 potentially eligible studies were retrieved for full text assessment and ineligible studies excluded with reason. No additional references were identified from citation searching (Figure 1). Finally, six studies reported in seven publications fulfilled the inclusion criteria and were included in the review.

### 3.2. Characteristics of Included Studies

Characteristics of included studies are shown in Table 2. Three studies were carried out in the United States (US) [40,41,42], two in Australia (three reports) [43,44,45], and one study in Canada [46]. Participants were individually randomised in all included studies. Five of the studies used a two-arm design while one study [46] employed a three-arm RCT design. Most of the included studies were small-scale explorations designed to assess the intervention acceptability and feasibility in larger-scale studies (Table S3). The number of participants ranged from 15 [42] to 83 [44] (Table S2), while the median sample size was 47. The mean age of participants varied across studies with means approximately ranging from 38 [41] to 62 years old [45]. Two studies (three reports) each involved postmenopausal women [40,44,45] or women with stage I–III breast cancer [44,45,46]. Among studies from the US one study included premenopausal women with history of gestational diabetes mellitus (GDM) [42], while two other studies included overweight [40] or obese women [41]. Interventions delivered in the included studies targeted participants with different activity levels including women who were inactive or insufficiently active and moderately active participants. Intervention duration lasted from 6 to 20 weeks, and follow-up periods of up to four months or less were reported.

The summary of intervention characteristics for all included studies is shown in Table 2. Intervention types varied across the studies but broadly included personal health tracking digital technologies. One of the included studies involved a novel sensor device with web portal and smartphone app software designed to assist in performance and compliance of pelvic floor muscle exercises [43]. The other five interventions (six reports) involved participants use of wearable activity monitoring devices to track and record physical activity levels [40,41,44,45,46] or sleep duration and efficiency [42]. Two studies [42,44] were supplemented with a feedback and goal setting sessions further enhanced by telephone delivered behavioural coaching sessions. Similarly, the study by Cadmus-Bertram et al. [40] focused on promoting physical activity self-monitoring/self-regulating skills among participants. In the study by McNeil et al. [46], participants received a diary with questions on goal setting, feasibility of prescribed physical activity targets, and strategies and barriers to physical activities participation which facilitated follow-up discussions between participants and the study exercise physiologist. The study by Joseph et al. [41] was a culturally tailored intervention for African American women which featured culturally tailored video and text-based physical activity promotion modules and online discussion board in addition to wearable activity monitors. The interventions in all studies were compared with alternative monitoring techniques or interventions [40,41,42,43], delayed intervention [44,45] or no intervention [46].

### 3.3. Risk of Bias Assessment of Included Studies

The overview of risk of bias of the included studies is presented in Table 3 and Table S4.

Risk of bias assessment showed that the randomisation domain was rated as having low risk of bias in three studies (four reports) [40,41,44,45], high risk in one study [43], while two studies had some concerns [42,46]. There was some concern regarding deviations from intended interventions in three studies (four reports) [40,44,45,46], one study was judged to have high risk [43] and two studies had low risk [41,42]. Two studies [43,46] were judged to have high risk of bias due to lack of information on missing outcome data for study participants. Some outcome measures were based on self-report in four studies [41,42,43,45], while there was no indication that outcome assessors were blinded to intervention groups in five the studies (six reports) [40,42,43,44,45,46]. Two studies [40,44] did not report sufficient information on selection of reported results and was judged to have a high risk of bias, while two studies [42,43] had some concern with the selection of reported results. Overall bias assessment was judged to be high in five studies [40,42,43,44,45,46] and low in only one study [41].

### 3.4. Effects of Interventions

In this review, intervention effects were categorised into three outcome groups: health status, well-being or quality of life (primary outcomes) and behaviour change (secondary outcome) (Table 3 and Table S5). The most commonly reported intervention effects were behaviour change, reporting changes in physical activity levels [40,41,44,45,46] and sleep patterns [42,46].

Two reports from one study [44,45] examined the effect of a wearable technology activity monitor coupled with behaviour feedback on sedentary behaviour and health related quality of life and fatigue in breast cancer survivors. Two studies [41,46] aimed to increase physical activity level and improve health-related outcomes using activity tracker. The study by Joseph et al. [41] involved African American women with obesity and assessed the effect of a culturally tailored smartphone-delivered physical activity intervention on changes in level of physical activity and cardiometabolic risk markers. McNeil et al. [46] used a wrist worn activity tracker to prescribe different physical activity intensities among breast cancer survivors to reduce sedentary time, and improve health outcomes. One study explored the feasibility of technology-assisted behavioural sleep extension using wearable sleep tracker in women with a history of GDM and short sleep [42]. Edwards et al. [43] assessed the effect of a novel sensor device with Web Portal and smartphone app designed to assist in the performance of and compliance with pelvic floor muscle exercise in female with urinary incontinence. The summary of intervention types and overall effects is presented in Table 3.

#### 3.4.1. Health Status

Outcomes relating to health-related fitness were reported by three studies but varied in terms of the outcomes assessed. Outcomes assessed in the studies included cardiorespiratory fitness (VO_2_max), body mass index (BMI) or weight change [42,46], cardiometabolic risk markers [41], and blood glucose [42]. Significant increase in VO_2_max were noted at 12 weeks among the physical activity intervention participants given a wrist-worn device to record heart rate and physical activity intensity and duration throughout the intervention [46]. An increase in VO_2_max was still noted at 24 weeks; however, these changes were not significantly different than that seen in the control group. No significant changes in body weight or fat mass were reported between baseline and follow up among breast cancer survivors using activity trackers [46] or women with history of GDM and insufficient sleep assigned to the wearable sleep tracker group [42]. Though not statistically significant, Joseph et al. [41] found clinically relevant between-arm differences across groups wherein intervention participants using the smartphone app-delivered physical activity intervention showed greater improvements in cardiorespiratory fitness, systolic blood pressure, diastolic blood pressure and pulse wave velocity from baseline to 4 months. However, the study found no difference in BMI, inflammatory markers of cardiometabolic health at 4 months [41].

#### 3.4.2. Well-Being or Quality of Life

Three studies reported outcomes related to well-being or quality of life [42,43,45]. Vallance et al. [45] measured health-related quality of life among post-treatment stage I–III breast cancer patients enrolled in a trial that examined the efficacy of a wearable-based intervention to increase physical activity and reduce sedentary behaviours. Statistically significant between group differences were reported whereby the intervention group had a 4.6-point difference in fatigue scores at 12 weeks (95% confidence interval (CI): 1.3, 7.8) indicating improvement in fatigue profiles in the intervention group [45]. No significant differences were observed between groups on the other health-related quality of life variables [45]. In another study, intervention group participants using a wearable sleep tracker had decreased fatigue scores compared to study controls, while no differences were reported between groups for other well-being or quality of life outcomes [42]. Compared to baseline, Edwards et al. [43] reported improvements in quality of life among females with stress, or stress predominant urinary incontinence randomised to either pelvic floor muscle exercise only or sensor device and pelvic floor muscle exercise groups [43].

#### 3.4.3. Behaviour Change

Outcomes relating to behaviour change were reported in almost all the included studies. Five studies reported changes in various levels of physical activity [40,41,42,44,46] while two studies assessed changes in sleep time [42,46]. Two studies [44,46] found that the wearable technology-based physical activity intervention favoured the intervention group, whereby the intervention achieved objectively measured increases in moderate-to-vigorous physical activity [44,46] while at the same time reducing total and prolonged sitting time [44] or sedentary time [46]. In another study, premenopausal women receiving a wearable technology-assisted sleep intervention self-reported statistically significant increases in physical activity compared to controls [42]. Similar self-reported increase in moderate-to-vigorous physical activity was also reported among participants in a smartphone-delivered physical activity intervention, although there was no observed difference on the accelerometer-measured moderate-to vigorous physical activity [41]. There were also no statistically significant differences between intervention and control groups reported for other objectively measured behaviour change outcomes including time spent standing (min/d), number of sit–stand transitions, number of daily steps [44], and sleep duration [42,46] and efficiency [42]. Alternatively, Cadmus-Bertram et al. [40] compared two different wearable activity trackers and reported increased moderate-to-vigorous physical activity (in bouts and total) and increased steps per day in the web-based tracking group compared to the pedometer group [40].

## 4. Discussion

This systematic review aimed to synthesise the evidence on the effectiveness of different forms of IoT for improving health outcomes in non-pregnant women living in high-income countries. The current review is the first comprehensive synthesis to focus exclusively on non-pregnant women in high-income countries, addressing a critical gap in the literature. We included only RCT or cluster-RCT studies examining the role of apps and the IoT in improving various aspects of women’s health in this review. We identified six unique studies in seven publications that met the review inclusion criteria. Intervention types varied from wearable devices for tracking physical activity, calories, sleep, and rest time to sensor device with web portal and smartphone application for pelvic floor muscle exercise. The most frequently evaluated intervention was a wrist-worn device for monitoring physical activity patterns and intensity. While some interventions showed significant positive effect for behaviour change, there was a lack of consistent evidence for improving health status, well-being, or quality of life outcomes. Follow-up periods varied across the studies ranging, from four weeks to four months, while publication dates were between the year 2015 to 2023. Although some interventions showed promising effects, it is difficult to draw firm conclusions about the use of apps and IoT for improving women’s health due to limited evidence and heterogeneity in study population and design.

Five of the included studies reported health-related and well-being or quality of life related outcomes. However, the outcomes assessed varied widely across individual studies. Two studies [43,45] that assessed health-related quality of life across different domains had inconsistent findings, reporting significantly positive effects in only one domain among breast cancer survivors [45]. According to previous studies, excess body weight, current smoking and insufficient physical activity are among health behaviour factors associated with poor quality of life among patients undergoing breast cancer treatment [47]. Our findings from this review highlight the potential benefit of IoT interventions for promoting behaviour change. Furthermore, the association between long-term quality of life and fatigue has been established among breast cancer survivors [48]. In the current review, we found evidence to support the association between use of IoT interventions involving wearable technology-based activity monitors, and improvements in fatigue profiles [45] and cardiopulmonary fitness [46].

In relation to the secondary outcomes, four of the five included studies reported on behaviour change related outcomes, including increasing physical activity, reducing sedentary time and improving sleep quality. Among interventions aimed at improving physical activity, statistically significant improvements in moderate-to-vigorous physical activity levels were reported among breast cancer survivors [44,46] and African American women with obesity [41]. The use of wearable technology-based activity tracker provides data based on objective measures of physical activity and sedentary time proving to be valid and reliable tools for measuring and prescribing activity patterns. One of the included studies used a wearable activity tracker to prescribe and monitor different physical activity intensities for breast cancer survivors and showed significant reductions in sedentary time reported among the low-intensity group [46]. A recent viewpoint argued that wearable technology-based devices may be able to facilitate behaviour change through a better understanding of individual preferences, frequency, intensity and duration of prescribed activity based on real-time data [49].

Our review aimed to focus on various IoT used in healthcare, including functions such as technology for relaying medical information through mobile health or telemedicine, collecting data and monitoring. The prominent use of wearable IoT interventions in the included studies highlights the growing application of IoT devices for remote patient monitoring and self-management outside of traditional healthcare settings. In addition to the wearable-based IoT interventions, one study included telephone-delivered behavioural counselling [44], while another included text-based physical activity promotion messages and an online discussion board forum [41]. Previous studies have demonstrated the effectiveness of telephone-based care for improving health behaviour [50], self-health management and quality of life among non-pregnant women [51]. Similarly, the use of real-time data sharing of IoT devices along with secure messaging systems that promote direct communication and personalised feedback with healthcare providers may provide unique opportunities for improving women’s health.

The certainty of evidence presented in this narrative summary may be weakened due to methodological variation and differences in the outcome assessment methods. We had planned to perform a meta-analysis for the primary and secondary outcomes in order to compare individual IoT interventions. However, no meta-analysis was performed due to variations in outcomes reported on a particular topic which limits the possibility of evidence synthesis, thus making it difficult to make decisions in practice. Also noteworthy is the fact that most studies included in this review were pilot phase intervention trials designed to assess the acceptability and feasibility of wearable or sensor-based IoT interventions. While included studies assessed acceptability among study participants, user acceptance among healthcare professionals has also been identified as an important challenge [52]. Although past research suggests that women find IoT devices such as wearables comfortable, convenient, affordable, and effective [22], more large-scale studies are needed to clearly demonstrate intervention effects, safety and scalability of the IoT in women’s healthcare.

The current review has several limitations that must be highlighted. Heterogeneity was high among the included studies, thereby precluding the possibility of conducting a meta-analysis. This was further exacerbated by the multi-component nature of the interventions considered in this review as previously highlighted [49,53]. There was a wide variation across studies and settings in defining what constitutes IoT interventions. The lack of a universally agreed-upon definitions and terminologies for IoT in healthcare creates challenges in determining what constitutes an IoT intervention. While the findings from this systematic review may suggest some positive effect, the single effect of IoT interventions among included studies remains unclear. Despite the rapid growth in the use of IoT worldwide, our review only focused on women living in high-income countries. This focus was deliberate and justified to reduce heterogeneity. As the disease profile and burden among women attracting IoT interventions may differ across high- and low- and middle-income countries, we focused on high-income countries in an effort to reduce heterogeneity in the objectives and settings of IoT interventions. Hence, our findings may not be generalisable to women outside this region.

## 5. Conclusions

This systematic review aimed to synthesise the role of apps and the IoT in improving the health of non-pregnant women living in high-income countries. Based on our findings, while it is indicative that IoT interventions, particularly wearable technologies, show potential for promoting behaviour change and improving specific health-related outcomes among non-pregnant women in high-income countries, the overall evidence remains inconclusive. Our review highlights both the promise of IoT for improving health in non-pregnant women, and the challenges posed by methodological inconsistencies and variations in outcome assessments across included studies. Most of the included studies were pilot studies, and the lack of standardised definitions for IoT interventions underscore the need for larger, more rigorously designed trials. Future research should aim to address these gaps, prioritising well-designed RCTs with larger sample sizes to ensure a clearer understanding of the effectiveness, acceptability, and scalability of IoT applications in women’s healthcare. Although the use of Apps and IoT interventions show some promise for facilitating behaviour change and improving certain aspects of health for non-pregnant women, stronger evidence is needed to fully advance our knowledge in this area for more tailored and effective health interventions for improving the health status, well-being, and quality of life among non-pregnant women.

## Figures and Tables

**Table 1:** PICOS framework showing review eligibility criteria.

	Inclusion Criteria	Exclusion Criteria
Population (P)	Non-pregnant working-aged women Women living in high-income countries	Studies including men only Studies including male and female population where outcome data is not separated by gender Studies of mixed population with <80% female participants
Intervention (I)	IoT interventions including applications, smartphones, and wearable devices used to improve women’s health	IoT interventions targeting pregnancy and postpartum period only
Comparison (C)	Standard care No intervention Other interventions not utilising IoT	NA
Outcome (O)	Primary outcomes Health status including number of cases diagnosed or treated Well-being Quality of life	Outcomes during pregnancy and postpartum period only
	Secondary outcome Lifestyle and behavioural changes	
Study design (S)	Individual randomised controlled trials (RCTs) and cluster-RCTs Studies reported in English language Studies conducted in high-income settings	Review articles Qualitative studies Observational studies including cross-sectional studies, case studies Commentaries, editorials, expert opinions, and letters

**Table 2:** Characteristics of included studies.

Study ID (Country)	Study Period	Study Design, Sample Size	Participant	Intervention(s)	Duration	Comparison
Lynch et al. [44] Vallance et al. [45] (Australia)	July 2016–July 2017	Two-arm individual RCT N = 83 (Intervention = 43; Control = 40)	Inactive postmenopausal women diagnosed with stage I–III breast cancer who had completed primary treatment Mean age: 61.6 ± 6.4	Wearable technology activity monitor (Garmin Viofit 2) Behavioural feedback and goal-setting session Telephone-delivered behavioural counselling	12 weeks; follow-up 12 weeks later	Delayed intervention
Cadmus-Bertram et al. [40] (USA)	2013–2014	Two-arm individual RCT N = 51 (Intervention 25; Control 26)	Participants were overweight postmenopausal women performing 60 min/week of MVPA Mean age: 60.0 ± 7.1	A low-touch, Fitbit-based PA intervention focused on self-monitoring/self-regulation skills	16 weeks; follow-up 4 weeks later	Provision of a basic step-counting pedometer
Edwards et al. [43] (Australia)	Not described	Two-arm individual RCT N = 22 (Number of people in each group not reported)	Females aged ≥18 years with stress, or mixed with predominantly stress, urinary incontinence Mean age: 42.5	PeriCoach System and PFME	20 weeks	PFME
McNeil et al. [46] (Canada)	February 2017–April 2018	Single centre three armed RCT N = 45 (Interventions 15, 15; Control 15)	Women 18 years or older who have been diagnosed with stage I–IIIc breast cancer and have completed adjuvant treatment Mean age: 60.0 ± 9.0	Lower or higher-intensity PA. A wrist-worn Polar A360^®^ device to record HR/PA intensity and PA duration throughout the intervention	12 weeks; follow-up 12 weeks later	No intervention
Joseph et al. [41] (USA)	January 2019–August 2019	Two-arm individual RCT N = 60 (Intervention 30; Control 30)	Insufficiently active African American women with obesity aged 24–49 years Mean age: 38.4 ± 6.9	Smart Walk smartphone-delivered PA intervention. The Smart Walk app included four key features: Personal profile pages Culturally tailored video and text-based PA promotion module Online discussion board forums PA self-monitoring feature that integrated with Fitbit activity monitors	4 months; follow-up 4 months later	Surface-level, culturally tailored health promotion intervention without PA tracking tool, using the same smartphone application platform as the intervention group
Reutrakul et al. [42] (USA)	February 2019–July 2021	Two-arm individual RCT N = 15 (intervention 9; control 6)	Premenopausal women aged 18–45 years with a history of GDM Mean age = 38.7–42.0	Fitbit wearable sleep tracker, with data accessible to the coach for guidance Fitbit smartphone application offering interactive feedback and tools Weekly didactic content via email on topics such as healthy sleep education Weekly brief telephone coaching sessions for reinforcement of didactic content, feedback based on sleep tracker data, progress review, barrier troubleshooting, and goal setting for the following week	6 weeks	Weekly health education emails and brief weekly telephone contact with the coach

PA—physical activity; RCT—randomised controlled trial; MVPA—moderate to vigorous physical activity; PFME—pelvic floor muscle exercises; HR—heart rate; GDM—gestational diabetes mellitus.

**Table 3:** Effect of intervention and risk of bias of included studies.

Study ID	Intervention	Intervention Effect Between Groups	Primary Outcomes		Secondary Outcomes	Overall Risk of Bias
			Health Status	Well-Being or Quality of Life	Behaviour Change	
Lynch et al. [44] Vallance et al. [45]	Wearable technology activity monitor coupled with a behavioural feedback and goal-setting session and telephone-delivered behavioural counselling	Significant positive effect			Sasaki MVPA (≥2690 cpm, triaxial)	High
					Sasaki MVPA bouts (≥2690 cpm, triaxial)	
					Freedson MVPA (≥1952 cpm, uniaxial)	
					Freedson MVPA bouts (≥1952 cpm, uniaxial)	
					Matthews MVPA bouts (≥760 cpm, uniaxial)	
					Sitting time, min/day	
					Sitting time bouts, min/day	
		No significant difference			Matthews MVPA (≥760 cpm, uniaxial)	
					Standing time	
					No. of sit-to-stand transitions	
					No. of steps	
		Significant positive effect		QOL: FACIT-Fatigue score (0–52)		
		No significant difference		QOL: FACT-B Breast cancer sub-scale (0–40)		
				QOL: FACT-B trial outcome index (0–96)		
				QOL: FACT-B General (0–108)		
				QOL: FACT-B total (0–148)		
Cadmus-Bertram et al. [40]	Fitbit-based PA intervention focused on self-monitoring/self-regulation skills	No significant difference			min/week moderate to vigorous intensity PA (total)	High
					min/week moderate to vigorous intensity PA (in bouts)	
					min/week light intensity PA	
					Average steps/day	
Edwards et al. [43]	Sensor device			Incontinence QOL		High
McNeil et al. [46]	Wrist-worn Polar A360^®^ device to record HR/PA intensity and PA duration throughout prescribed 300 min/week of lower-intensity PA or 150 min/week of higher-intensity PA	Significant positive effect	Cardiorespiratory fitness VO_2_max		Moderate-vigorous intensity PA time (min/day)	High
					Sedentary time (min/day)	
		No significant difference	BMI (kg/m^2^)		Total PA time (min/day)	
					Light-intensity activity time (min/day)	
					Sleep time (min/day)	
Joseph et al. [41]	Smart Walk smartphone app-delivered PA intervention—Fitbit Inspire HR activity monitor	Significant positive effect			Self-reported MVPA (min/week)	Low
		No significant difference	Systolic Blood Pressure (mmHG)		Accelerometer-measured MVPA (min/day)—1 min bouts	
			Diastolic Blood Pressure (mmHG)		Accelerometer-measured MVPA (min/day)—10 min bouts	
Reutrakul et al. [42]	Fitbit wearable sleep tracker	Significant positive effect		Promis fatigue T-score	IPAQ (MET-min/week)	High
		No significant difference	Fasting glucose (mg/dL)	PSQI	Sleep duration (min)	
			2 h glucose (mg/dL)	GAD-7 score	Sleep efficiency (%)	
			Weight change (kg)	CES-D		

MVPA—moderate to vigorous physical activity; FACIT-Fatigue—Functional Assessment of Chronic Illness Therapy-Fatigue; FACT-B—Functional Assessment of Cancer Therapy-Breast; QOL—Quality of life; PA—physical activity; HR—heart rate; BMI—body mass index; IPAQ—International Physical Activity Questionnaire; PSQI—Pittsburgh Sleep Quality Index; GAD-7—General Anxiety Disorder-7; CES-D—Center for Epidemiologic Studies Depression Scale.

## Data Availability

No new data were created or analyzed in this study.

## Supplementary Materials

The following supporting information can be downloaded at: https://www.mdpi.com/article/10.3390/healthcare13243310/s1, Supplementary Material S1: Search strategy; Table S1: PRISMA Checklist and Abstracts Checklist; Table S2: Search resources details and number of results; Table S3: Description of study phase of included studies; Table S4: Risk of Bias for included studies; Table S5: Intervention effect summary from included studies.

## Abbreviations

The following abbreviations are used in this manuscript:

Apps	Digital applications
IoT	Internet of Things
HIC	High-income countries
WHO	World Health Organisation
PRISMA	Preferred Reporting Items for Systematic Reviews and Meta-Analyses
CINAHL	Cumulative Index to Nursing and Allied Health Literature
PICOS	Population, intervention, comparison, outcome, and study design
RCT	Randomised controlled trials
US	United States
GDM	Gestational diabetes mellitus
BMI	Body mass index
CI	Confidence interval
