# Supplementary material for: Effectiveness of the Internet of Things for Improving Health of Non-Pregnant Women Living in High-Income Countries: A Systematic Review
Source: Healthcare (Basel). 2025 Dec 17;13(24):3310. doi: 10.3390/healthcare13243310 (PMC12733010; doi:10.3390/healthcare13243310)
Supplement: Supplementary file 1 [file healthcare-13-03310-s001.zip › Tables S1--S5.pdf]

Table S1. PRISMA Checklist

Section and Topic	Item #	Checklist item	Location where item is reported
**Title**			
Title	1	Identify the report as a systematic review.	Pg 1
**Abstract**			
Abstract	2	See the PRISMA 2020 for Abstracts checklist.	Abstract
**Introduction**			
Rationale	3	Describe the rationale for the review in the context of existing knowledge.	Pg 2, 3
Objectives	4	Provide an explicit statement of the objective(s) or question(s) the review addresses.	Pg 3
**Methods**			
Eligibility criteria	5	Specify the inclusion and exclusion criteria for the review and how studies were grouped for the syntheses.	Pg 3, table 1
Information sources	6	Specify all databases, registers, websites, organisations, reference lists and other sources searched or consulted to identify studies. Specify the date when each source was last searched or consulted.	Pg 3
Search strategy	7	Present the full search strategies for all databases, registers and websites, including any filters and limits used.	Supplementary material S1
Selection process	8	Specify the methods used to decide whether a study met the inclusion criteria of the review, including how many reviewers screened each record and each report retrieved, whether they worked independently, and if applicable, details of automation tools used in the process.	Pg 4
Data collection process	9	Specify the methods used to collect data from reports, including how many reviewers collected data from each report, whether they worked independently, any processes for obtaining or confirming data from study investigators, and if applicable, details of automation tools used in the process.	Pg 4-5
Data items	10a	List and define all outcomes for which data were sought. Specify whether all results that were compatible with each outcome domain in each study were sought (e.g. for all measures, time points, analyses), and if not, the methods used to decide which results to collect.	Table 1
	10b	List and define all other variables for which data were sought (e.g. participant and intervention characteristics, funding sources). Describe any assumptions made about any missing or unclear information.	Pg 4-5
Study risk of bias assessment	11	Specify the methods used to assess risk of bias in the included studies, including details of the tool(s) used, how many reviewers assessed each study and whether they worked independently, and if applicable, details of automation tools used in the process.	Pg 5

Effect measures	12	Specify for each outcome the effect measure(s) (e.g. risk ratio, mean difference) used in the synthesis or presentation of results.	Pg 5
Synthesis methods	13a	Describe the processes used to decide which studies were eligible for each synthesis (e.g. tabulating the study intervention characteristics and comparing against the planned groups for each synthesis (item #5)).	Pg 5
	13b	Describe any methods required to prepare the data for presentation or synthesis, such as handling of missing summary statistics, or data conversions.	Pg 5
	13c	Describe any methods used to tabulate or visually display results of individual studies and syntheses.	Pg 5
	13d	Describe any methods used to synthesize results and provide a rationale for the choice(s). If meta-analysis was performed, describe the model(s), method(s) to identify the presence and extent of statistical heterogeneity, and software package(s) used.	Pg 5
	13e	Describe any methods used to explore possible causes of heterogeneity among study results (e.g. subgroup analysis, meta-regression).	Pg 5
	13f	Describe any sensitivity analyses conducted to assess robustness of the synthesized results.	Pg 5
Reporting bias assessment	14	Describe any methods used to assess risk of bias due to missing results in a synthesis (arising from reporting biases).	Pg 5
Certainty assessment	15	Describe any methods used to assess certainty (or confidence) in the body of evidence for an outcome.	Pg 5
**Results**			
Study selection	16a	Describe the results of the search and selection process, from the number of records identified in the search to the number of studies included in the review, ideally using a flow diagram.	Pg 6
	16b	Cite studies that might appear to meet the inclusion criteria, but which were excluded, and explain why they were excluded.	Figure 1
Study characteristics	17	Cite each included study and present its characteristics.	Pg 6, table 2
Risk of bias in studies	18	Present assessments of risk of bias for each included study.	Pg 8, Supplementary material Table S4
Results of individual studies	19	For all outcomes, present, for each study: (a) summary statistics for each group (where appropriate) and (b) an effect estimate and its precision (e.g. confidence/credible interval), ideally using structured tables or plots.	Pg 10, Supplementary material Table S5
Results of syntheses	20a	For each synthesis, briefly summarise the characteristics and risk of bias among contributing studies.	Table 3
	20b	Present results of all statistical syntheses conducted. If meta-analysis was done, present for each the summary estimate and its precision (e.g. confidence/credible interval) and measures of statistical heterogeneity. If comparing groups, describe the direction of the effect.	Table 3, pg 10-11

	20c	Present results of all investigations of possible causes of heterogeneity among study results.	N/A
	20d	Present results of all sensitivity analyses conducted to assess the robustness of the synthesized results.	N/A
Reporting biases	21	Present assessments of risk of bias due to missing results (arising from reporting biases) for each synthesis assessed.	N/A
Certainty of evidence	22	Present assessments of certainty (or confidence) in the body of evidence for each outcome assessed.	N/A
**Discussion**			
Discussion	23a	Provide a general interpretation of the results in the context of other evidence.	Pg 12
	23b	Discuss any limitations of the evidence included in the review.	Pg 13
	23c	Discuss any limitations of the review processes used.	Pg 13
	23d	Discuss implications of the results for practice, policy, and future research.	Pg 13
**Other information**			
Registration and protocol	24a	Provide registration information for the review, including register name and registration number, or state that the review was not registered.	Pg 3
	24b	Indicate where the review protocol can be accessed, or state that a protocol was not prepared.	Pg 3
	24c	Describe and explain any amendments to information provided at registration or in the protocol.	Pg 5
Support	25	Describe sources of financial or non-financial support for the review, and the role of the funders or sponsors in the review.	Financial disclosure section of the submission system
Competing interests	26	Declare any competing interests of review authors.	Competing interests' section of submission system
Availability of data, code and other materials	27	Report which of the following are publicly available and where they can be found: template data collection forms; data extracted from included studies; data used for all analyses; analytic code; any other materials used in the review.	N/A

Abstract Checklist

Section and Topic	Item #	Checklist item	Reported (Yes/No)
**Title**			
Title	1	Identify the report as a systematic review.	Yes
**Background**			
Objectives	2	Provide an explicit statement of the main objective(s) or question(s) the review addresses.	Yes
**Methods**			
Eligibility criteria	3	Specify the inclusion and exclusion criteria for the review.	Yes
Information sources	4	Specify the information sources (e.g. databases, registers) used to identify studies and the date when each was last searched.	Yes
Risk of bias	5	Specify the methods used to assess risk of bias in the included studies.	Yes
Synthesis of results	6	Specify the methods used to present and synthesise results.	Yes
**Results**			
Included studies	7	Give the total number of included studies and participants and summarise relevant characteristics of studies.	Yes
Synthesis of results	8	Present results for main outcomes, preferably indicating the number of included studies and participants for each. If meta-analysis was done, report the summary estimate and confidence/credible interval. If comparing groups, indicate the direction of the effect (i.e. which group is favoured).	Yes
**Discussion**			
Limitations of evidence	9	Provide a brief summary of the limitations of the evidence included in the review (e.g. study risk of bias, inconsistency and imprecision).	Yes
Interpretation	10	Provide a general interpretation of the results and important implications.	Yes
**Other**			
Funding	11	Specify the primary source of funding for the review.	No
Registration	12	Provide the register name and registration number.	Yes

Table S2: Search resources details and number of results

Resource	Time coverage	Search interface	# of hits
CENTRAL	Until Search Date	Cochrane Library	9197
CINAHL [Excluding MEDLINE]	1937 – Search Date	EBSCOhost	657
ClinicalTrials.gov	Until Search Date	ClinicalTrials.gov	1086
Embase	1974 – 2023 Week 06	Ovid SP	3021
Ovid MEDLINE(R) ALL	1946 – February 10, 2023	Ovid SP	2821
APA PsycINFO	1806 – January Week 5 2023	Ovid SP	504
PubMed [Excluding MEDLINE]	1946 – Search Date	PubMed	704
WHO ICTRP	Until Search Date	WHO ICTRP	433
Subtotal			18,433
Duplicates			4484
**Total (for Screening)**			**13949**

Table S3. Description of study phase of included studies

Study id	Intervention target	Type of wearable device	Sample size	Study phase
Lynch et al., 2019	Increase MVPA and reduce sedentary behaviour	Wrist-worn Garmin Vivofit2 activity monitor	83	Efficacy trial
Vallance et al., 2019	Changes in quality of life			
Cadmus-Bertram et al., 2015	Increase physical activity	Fitbit One worn on the waistband, bra or placed in the pocket	51	RCT to assess feasibility and preliminary efficacy of intervention
Edwards et al., 2016	Improve urinary continence; incontinence quality of life	PeriCoach - pelvic floor trainer device	22	Ongoing efficacy trial
Mcneil et al., 2019	Increase physical activity	Wrist-worn Polar A360® device	45	Pilot trial to develop the methods for and establish the feasibility of a full scale trial
Joseph et al., 2023	Increase physical activity and reduce cardiometabolic disease risk	Fitbit	60	Pilot trial to examine the feasibility and acceptability of the intervention
Reutrakul et al., 2022	Sleep extension	Fitbit®	15	Pilot trial to assess feasibility (e.g., acceptability and choice of appropriate outcomes of the future trial)

Table S4: Risk of Bias for included studies

	Domains					
**Author (year), country**	**1. Randomisation process**	**2. Deviations from intended interventions**	**3. Missing outcome data**	**4. Measurement of the outcome**	**5. Selection of reported result**	**6. Overall bias**
**Lynch et al. (2019), Australia**	**Low**	**Some concerns**	**Low**	**Low**	**High**	**High**
Increasing physical activity: Sasaki moderate to vigorous physical activity (MVPA) (≥2690 cpm, triaxial)	Low	Low	Low	Low	High	High
Increasing physical activity: Sasaki MVPA bouts (≥2690 cpm, triaxial)	Low	Low	Low	Low	High	High
Increasing physical activity: Freedson MVPA (≥1952 cpm, uniaxial)	Low	Low	Low	Low	High	High
Increasing physical activity: Freedson MVPA bouts (≥1952 cpm, uniaxial)	Low	Low	Low	Low	High	High
Increasing physical activity: Freedson MVPA boutsa (≥1952 cpm, uniaxial)	Low	Low	Low	Low	High	High
Increasing physical activity: Matthews MVPA bouts (≥760 cpm, uniaxial)	Low	Low	Low	Low	High	High
Increasing physical activity: activPAL measured sedentary behaviour Sitting time, min/d	Low	Some concerns	Low	Low	Low	Some concerns
Increasing physical activity: Sitting time bouts,b min/d	Low	Some concerns	Low	Low	Low	Some concerns
Increasing physical activity: Standing time, min/d	Low	Some concerns	Low	Low	Low	Some concerns
Increasing physical activity: No. of sit-to-stand transitions	Low	Some concerns	Low	Low	Low	Some concerns
Increasing physical activity: No. of steps	Low	Some concerns	Low	Low	Low	Some concerns
**Vallance et al. (2019), Australia**	**Low**	**Some concerns**	**Low**	**Some concerns**	**Low**	**Some concerns**
QOL: FACT-B Breast cancer subscale (0-40)	Low	Some concerns	Low	Some concerns	Low	Some concerns
QOL: FACT-B trial outcome index (0-96)	Low	Some concerns	Low	Some concerns	Low	Some concerns

QOL: FACT-General (0-108)	Low	Some concerns	Low	Some concerns	Low	Some concerns
QOL: FACT-B total (0-148)	Low	Some concerns	Low	Some concerns	Low	Some concerns
**Cadmus-Bertram et al. (2015), USA**	**Low**	**Some concerns**	**Low**	**Low**	**High**	**High**
Increasing physical activity: Moderate to vigorous intensity (total)	Low	Some concerns	Low	Low	High	High
Increasing physical activity: Moderate to vigorous intensity (in bouts)	Low	Some concerns	Low	Low	High	High
Increasing physical activity: Light intensity	Low	Some concerns	Low	Low	High	High
Increasing physical activity: Average steps per day	Low	Some concerns	Low	Low	High	High
**Edwards et al. (2016), Australia**	**High**	**High**	**High**	**High**	**Some concerns**	**High**
Quality of Life	High	High	High	High	Some concerns	High
**Mcneil et al. (2019), Canada**	**Some concerns**	**Some concerns**	**High**	**High**	**Low**	**High**
Total PA time at 12 wk	Some concerns	Some concerns	Low	Low	Low	Some concerns
Moderate-vigorous intensity PA time at 12 wk	Some concerns	Some concerns	Low	High	Low	High
Light-intensity activity time at 12 wk	Some concerns	Some concerns	Low	Low	Low	Some concerns
Sedentary time at 12 wk (min d)	Some concerns	Some concerns	Low	High	Low	High
Sleep time (min d) at 12 wk	Some concerns	Some concerns	Low	Low	Low	Some concerns
Body mass index (kg·m2) at 12 wk	Some concerns	Some concerns	Low	Low	Low	Some concerns
Cardiorespiratory fitness/ VO2max (mL·kg-1·min-1 at 12 wk	Some concerns	Some concerns	Low	Low	Low	Some concerns
Total PA time at 24 wk	Some concerns	Low	High	Low	Low	High
Moderate-vigorous intensity PA time at 24 wk	Some concerns	Low	High	Low	Low	High
Light-intensity activity time at 24 wk	Some concerns	Low	High	Some concerns	Low	High
Sedentary time at 24 wk (min d)	Some concerns	Low	High	High	Low	High
Sleep time (min d) at 24 wk	Some concerns	Low	High	Low	Low	High
Body mass index (kg·m 2) at 24 wk	Some concerns	Low	High	Low	Low	High

Cardiorespiratory fitness/ VO2max (mL·kg-1·min-1 at 24 wk	Some concerns	Low	High	Low	Low	High
**Joseph et al. (2023), USA**	**Low**	**Low**	**Low**	**Low**	**Low**	**Low**
Self-reported MVPA at 4 months f/u (mins./week)	Low	Low	Low	Low	Low	Low
Self-reported MVPA at 8 months f/u (mins./week)	Low	Low	Low	Low	Low	Low
Accelerometer-measured MVPA (1 min bouts) at 4 months f/u (mins./week)	Low	Low	Low	Low	Low	Low
Accelerometer-measured MVPA (1 min bouts) at 8 months f/u (mins./week)	Low	Low	Low	Low	Low	Low
Accelerometer-measured MVPA (10 mins bouts) at 4 months f/u (mins./week)	Low	Low	Low	Low	Low	Low
Accelerometer-measured MVPA (10 mins bouts) at 8 months f/u (mins./week)	Low	Low	Low	Low	Low	Low
Systolic Blood Pressure (mmHg) at 4 months	Low	Low	Low	Low	Low	Low
Diastolic Blood Pressure (mmHg) at 4 months	Low	Low	Low	Low	Low	Low
Systolic Blood Pressure (mmHg) at 8 months	Low	Low	Low	Low	Low	Low
Diastolic Blood Pressure (mmHg) at 8months	Low	Low	Low	Low	Low	Low
**Reutrakul et al. (2022), USA**	**Some concerns**	**Low**	**Low**	**High**	**Some concerns**	**High**
Sleep duration (minutes)	Some concerns	Low	Low	High	Some concerns	High
Sleep efficiency (%)	Some concerns	Low	Low	High	Some concerns	High
Fasting blood glucose (mg/dL)	Some concerns	Low	Low	Low	Some concerns	Some concerns
2h glucose (mg/dL)	Some concerns	Low	Low	Low	Some concerns	Some concerns
AUC glucose	Some concerns	Low	Low	Low	Some concerns	Some concerns
HOMA-IR	Some concerns	Low	Low	Low	Some concerns	Some concerns
Weight (kg)	Some concerns	Low	Low	High	Some concerns	High
PSQI scores	Some concerns	Low	Low	High	Some concerns	High
GAD-7 scores	Some concerns	Low	Low	High	Some concerns	High
Promis fatigue T-score	Some concerns	Low	Low	High	Some concerns	High

IPAQ (Met-minutes/week)	Some concerns	Low	Low	High	Some concerns	High
CES-D scores	Some concerns	Low	low	High	Some concerns	High

MVPA=moderate-to-vigorous physical activity; QOL=Quality of life; FACT-B= Functional Assessment of Cancer Therapy – Breast; PA=physical activity; HOMA-IR= Homeostatic Model Assessment for Insulin Resistance; PSQI=Pittsburgh Sleep Quality Index; GAD-7= General Anxiety Disorder-7; IPAQ= International Physical Activity Questionnaire; CES-D= Center for Epidemiologic Studies Depression Scale

Table S5. Intervention effect summary from included studies

Study ID	Intervention (# of participants)	Comparator	Analyses	Outcome	Results (95%CI)			Remarks
					Mean (SD)	Within arm change	Difference in change between arms	
Lynch et al. (2019); Vallance et al. (2019) Australia	Use of the Garmin Vivofit 2 activity monitor coupled with a behavioral feedback and goal-setting session and telephone-delivered health coaching sessions<br>n=83	Delayed intervention	Linear mixed model analy- sis was used to investigate within- and between-group changes in MVPA and sedentary behavior<br>Models were adjusted for valid accelerometer wear time and awake time<br>Sensitivity analyses in which participants with fewer than 4 valid days of accelerometer data were excluded	Actigraph measured MVPA, min/wk<br>Sasaki MVPA (≥2690 cpm, triaxial)	Intervention arm: 249.6 (159.7)<br>Control arm: 170.1 (127.9)	Intervention arm: 66.5 (33.3 to 99.8)<br>Control arm: −2.1 (−35.4 to 31.1)	68.7 (21.7 to 115.6)	Results from the ACTIVATE Trial suggest that the use of wearable technology presents an inexpensive and scalable opportunity to facilitate more active lifestyles for cancer survivors<br>Results from sensitivity analyses were not materially different from the results obtained utilizing all data available
				Sasaki MVPA bouts (≥2690 cpm, triaxial)	Intervention arm: 67.2 (77.4)<br>Control arm: 36.8 (62.0)	Intervention arm: 40.4 (22.0 to 58.8)<br>Control arm: 1.7 (−16.7 to 20.1)	38.7 (12.7 to 64.7)	
				Freedson MVPA (≥1952 cpm, uniaxial)	Intervention arm: 159.0 (127.2)<br>Control arm: 117.1 (109.9)	Intervention arm: 52.0 (24.4 to 79.6)<br>Control arm: 11.4 (−16.1 to 40.0)	40.6 (1.67 to 79.5)	
				Freedson MVPA bouts (≥1952 cpm, uniaxial)	Intervention arm: 56.3 (70.4)<br>Control arm: 35.8 (59.9)	Intervention arm: 33.8 (17.1 to 50.4)<br>Control arm: 5.6 (−11.0 to 22.18)	28.2 (4.7 to 51.7)	
				Matthews MVPA (≥760 cpm, uniaxial)	Intervention arm: 609.2 (313.9)<br>Control arm: 510.8 (235.0)	Intervention arm: 69.7 (6.5 to 132.8)<br>Control arm: 0.10 (−63.0 to 63.2)	69.6 (−19.6 to 158.7)	
				Matthews MVPA boutsa (≥760 cpm, uniaxial)	Intervention arm: 119.0 (114.9)<br>Control arm: 76.7 (93.4)	Intervention arm: 54.4 (25.5 to 83.3)<br>Control arm: 10.0 (−18.9 to 38.9)	44.4 (3.6 to 85.2)	

				activPAL measured sedentary behaviour Sitting time, min/d	Intervention arm: 530.0 (103.6)<br>Control arm: 553.8 (176.7)	Intervention arm: −23.5 (−49.0 to −2.0)<br>Control arm: 13.1 (−11.1 to 37.3)	−36.6 (−71.7 to −1.6)	
				Sitting time bouts, min/d	Intervention arm: 357.1 (117.0)<br>Control arm: 380.1 (184.4)	Intervention arm: −32.3 (−61.8 to −2.8)<br>Control arm: 9.8 (−18.2 to 37.8)	−42.1 (−82.7 to −1.6)	
				Standing time	Intervention arm: 244.9 (91.1)<br>Control arm: 265.1 (86.1)	Intervention arm: 11.5 (−9.8 to 32.9)<br>Control arm: −15.6 (−35.9 to 4.6)	27.2 (−2.2 to 56.5)	
				No. of sit-to-stand transitions	Intervention arm: 45.9 (10.7)<br>Control arm: 44.7 (11.5)	Intervention arm: 2.5 (−0.7 to 5.8)<br>Control arm: 0.2 (−2.9 to 3.4)	2.3 (−2.2 to 6.9)	
				No. of steps	Intervention arm: 8193 (3301)<br>Control arm: 7539 (3404)	Intervention arm: 1241 (408 to 2074)<br>Control arm: 308 (−486 to 1102)	933 (−215 to 2082)	
			Primary intervention effects on HRQoL and fatigue were evaluated by comparing intervention and waitlist group at T2 using repeated measures mixed effects models on intention-to-treat basis	HRQoL FACIT-Fatigue score (0-52)	Intervention: 38.0 (8.9)<br>Waitlist control: 37.7 (11.1)	Intervention arm: 5.1 (2.0, 8.2)<br>Control arm: 0.5 (−1.2, 2.1)	4.6 (1.3, 7.8)	Despite small improvements in fatigue profiles, no effects on HRQoL were observed
				FACT-B HRQoL Breast cancer sub-scale (0-40)	Intervention: 25.5 (5.8)<br>Waitlist control: 24.9 (4.7)	Intervention arm: 0.8 (−0.7, 2.3)<br>Control arm: 0.8 (−0.5, 2.0)	0.1 (−1.8, 2.0)	
				FACT-B HRQoL trial outcome index (0-96)	Intervention: 66.1 (16.2)<br>Waitlist control: 67.3 (13.4)	Intervention arm: 2.3 (−1.2, 5.9)<br>Control arm: 1.6 (−0.6, 3.8)	0.8 (−3.1, 4.7)	
				FACT-B HRQoL General (0-108)	Intervention: 77.8 (20.3)<br>Waitlist control: 80.5 (16.7)	Intervention arm: 2.4 (−1.9, 6.7)<br>Control arm: 1.1 (−1.5, 3.7)	1.3 (−3.4, 6.0)	
				FACT-B HRQoL total (0-148)	Intervention: 103.5 (24.5)<br>Waitlist control: 105.5 (20.2)	Intervention arm: 3.2 (−1.9, 8.2)<br>Control arm: 1.8 (−1.5, 5.2)	1.4 (−4.3, 7.1)	
Cadmus-Bertram et	A low-touch, Fitbit-based PA intervention focused on self-	Basic step-counting pedometer	Data analysed according to intention-to-treat ActiGraph data were	Minutes/week moderate to vigorous intensity PA (total)	Web-based tracking: 234 (119)<br>Pedometer: 189 (99)	Web-based tracking: 62 (108)<br>Pedometer: 13 (98)	0.48; p=0.11	In this study, a Fitbit-based intervention was

al. (2015) USA	monitoring/ self-regulation skills		adjusted for number of valid days<br>PA changes from baseline to Week 16 were assessed using repeated-measures ANCOVA, adjusted for age and ActiGraph daily wear time to address potential residual confounding<br>Missing data were imputed by carrying forward baseline values.	Minutes/week moderate to vigorous intensity PA (in bouts)	Web-based tracking: 62 (82)<br>Pedometer: 57 (69)	Web-based tracking: 38 (83)<br>Pedometer: 16 (76)	0.28; p=0.28	associated with increased PA, with no change observed in the Pedometer Group. Although between-group tests did not reach significance, this may be due to sample size
				Minutes/week light intensity PA	Web-based tracking: 1262 (320)<br>Pedometer: 1252 (317)	Web-based tracking: –14 (204)<br>Pedometer: –33 (225)	0.09; p=0.54	
				Average steps/day	Web-based tracking: 6695 (2708)<br>Pedometer: 6188 (2423)	Web-based tracking: 789 (1,979)<br>Pedometer: 362 (1,605)	0.24; p=0.3	
Edwards et al. (2016) Australia	PeriCoach System - a novel sensor device with Web Portal and Smartphone app software designed to assist in the performance of and compliance with PFME	PFME only		Incontinence Quality-of-Life	PFME/PC: 94.3 (9.3)<br>PFME: 81.3 (20.1)			Preliminary data shows that the PeriCoach may offer assistance in early improvement of urinary incontinence for those patients compliant with PFME. Both groups experienced improvements in measures of Quality of Life measures.
Mcneil et al. (2019) Canada	Prescription of 300 min/week of lower-intensity PA or 150 min/week of higher-intensity PA using activity trackers to increase PA	No PA	Repeated-measures ANOVA tests were also conducted to assess any changes across time.<br>Linear models estimated changes in study outcomes within each group at 12 and 24 wk, compared with baseline, after adjusting for baseline outcome values.	Total PA time (min/day)	Low intensity PA: 414 (90)<br>Higher intensity PA: 360 (78)<br>Control: 348 (96)	Low intensity PA: 66 (36 to 102)<br>Higher intensity PA: 42 (6 to 78)<br>Control: 24 (12 to 60)	48 (-6 to 96)<br>18 (-30 to 66)	Increases in PA time and cardiopulmonary fitness/VO2max can be achieved with both lower- and higher-intensity PA interventions in breast cancer survivors. Reductions in
				Moderate-vigorous intensity PA time (min/day)	Low intensity PA: 138 (36)<br>Higher intensity PA: 114 (48)<br>Control: 96 (48)	Low intensity PA: 42 (24 to 60)<br>Higher intensity PA: 24(6 to 42)<br>Control: 6 (-12 to 30)	36 (6 to 60)<br>12 (-12 to 42)	

				Light-intensity activity time (min/day)	Low intensity PA: 276 (72)<br>Higher intensity PA: 246 (54)<br>Control: 246 (66)	Low intensity PA: 24(3to48)<br>Higher intensity PA: 18 (-3 to 42)<br>Control: 12 (-6 to 36)	12 (-18 to 42)<br>6 (-24 to 36)	sedentary time were also noted in the lower-intensity PA group
				Sedentary time (min/day)	Low intensity PA: 534 (72)<br>Higher intensity PA: 576 (84)<br>Control: 594 (96)	Low intensity PA: -54 (-96 to -18)<br>Higher intensity PA: -6 (-48 to 30)<br>Control: 12 (-30 to 60)	-72 (-132 to -12)<br>-24 (-84 to 36)	
				Sleep time (min/day)	Low intensity PA: 432 (108)<br>Higher intensity PA: 480 (102)<br>Control: 456 (108)	Low intensity PA: -48 (-90 to -0.3)<br>Higher intensity PA: -12 (-60 to 36)<br>Control: -42 (-90 to 6)	-6 (-72 to 66)<br>30 (-36 to 102)	
				BMI (kg/m2)	Low intensity PA: 28.6 (5)<br>Higher intensity PA: 31 (8.5)<br>Control: 26 (3.8)	Low intensity PA: -0.1 (0.7 to 0.5)<br>Higher intensity PA: -0.02 (0.6 to 0.6)<br>Control: 0.3 (0.3 to 1.0)	0.4 (-1.3 to 0.4)<br>-0.4 (-1.2 to 0.5)	
				Cardiorespiratory fitness VO2max	Low intensity PA: 24 (7)<br>Higher intensity PA: 23.1 (9.2)<br>Control: 18.3 ((6.7)	Low intensity PA: 4.5 (2.0 to 7.0)<br>Higher intensity PA: 5.6 (3.1 to 8.2)<br>Control: 0.3 (-2.5 to 3.0)	4.2 (0.5 to 8.0)<br>5.4 (1.7 to 9.1)	
Joseph et al. (2023) USA	A culturally tailored PA promotion intervention delivered through the Smart Walk smartphone app and text messages	Smart Health smartphone-delivered attention-matched wellness	Intervention effects and effect sizes (Cohen's d) were estimated using ANCOVA-type multivariable regression models predicting post-intervention (4- or 8-month follow-up) values on each outcome from study arm	Self-reported MVPA (min/week)	Intervention: 102.2 (73.5)<br>Comparison: 58.5 (56.6)	N/A	0.69 (0.16 to 1.22)	Intervention participants self-reported greater MVPA increases than comparison group participants at both post-intervention assessments. Meaningful, though not
				Accelerometer-measured MVPA (min/day) - 1 minute bouts	Intervention: 13.8 (21.4)<br>Comparison: 20.0 (15.9)	N/A	-0.39 (-0.91 to 0.13)	
				Accelerometer-measured MVPA (min/day) - 10 min bouts	Intervention: 1.3 (4.9)<br>Comparison: 1.4 (5.0)	N/A	-0.25 (-0.77 to 0.27)	

				Systolic Blood Pressure (mmHG)	Intervention: 130.8 (17.2)<br>Comparison: 135.2 (16.1)	N/A	-0.22 (-0.74 to 0.34)	statistically significant, improvements in cardiorespiratory fitness and blood pressure were observed among intervention participants.
				Diastolic Blood Pressure (mmHG)	Intervention: 79.1 (13.5)<br>Comparison: 84.0 (11.3)	N/A	-0.37 (-0.89 to 0.15)	
Reutrakul et al. 2022	Fitbit wearable sleep tracker	Health education emails and brief telephone contact from the coach provided weekly (≤ 5 min)	Exploratory analyses to quantify efect sizes of outcomes between Sleep-Extend and healthy living control (i.e., means, standard deviations (SDs)). Diferences between groups (baseline characteristics and changes between baseline and end of intervention) were compared using independent samples t-tests or Mann Whitney U tests as appropriate	Sleep duration (minutes)	Sleep-extend: 26.9 (42.5)<br>Healthy living: − 9.1 (20.4)		35.9 (− 8.6, 80.5)	The study demonstrated that technology-assisted behavioural sleep extension was feasible in women with a history of GDM, with benefits in fatigue and physical activity, and possibly glucose metabolism.
				Sleep efficiency (%)	Sleep-extend: − 3.0 (3.1)<br>Healthy living: − 0.6 (1.9)		-2.4 (-5.7 to 0.9)	
				Fasting blood glucose (mg/dL)	Sleep-extend: 1.6 (9.4)<br>Healthy living: 10.4 (8.2)		− 8.8 (-19.8 to 2.1)	
				2h glucose (mg/dL)	Sleep-extend: 8.7 (20.6)<br>Healthy living: 10.4 (27.3)		− 1.6 (-29.6 to 26.4)	
				AUC glucose	Sleep-extend: 13.5 (35.1)<br>Healthy living: 6.6 (46.7)		6.9 (− 40.9 to 54.8)	
				HOMA-IR	Sleep-extend: 0.70 (1.40)<br>Healthy living: 0.59 (1.08)		0.11 (− 1.51 to 1.74)	
				Weight (kg)	Sleep-extend: − 0.22 (1.9)<br>Healthy living: − 1.66 (2.08)		1.43 (− 0.97 to 3.85)	
				PSQI scores	Sleep-extend: − 2.8 (3.2)<br>Healthy living: − 2.0 (4.2)		− 0.8 (− 5.1 to 3.5)	
				GAD-7 scores	Sleep-extend: 1.0 (− 1.5 to 2.0)<br>Healthy living: 1.0 (0.0 to 2.0)			
				Promis fatigue T-score	Sleep-extend: − 5.1 (8.7)<br>Healthy living: 5.5 (3.1)		− 10.6 (− 20.7 to − 0.6)	

	IPAQ (Met-minutes/week)	Sleep-extend: +1847 (3200)<br>Healthy living: − 3188 (4341)	5036 (343 to 9729)
	CES-D scores	Sleep-extend: − 1.9 (5.6)<br>Healthy living: +3.4 (3.8)	− 5.2 (-11.4 to 0.9)

MVPA=moderate-to-vigorous physical activity; HRQoL=Health-related quality of life; FACIT-Fatugue score= Functional Assessment of Chronic Illness Therapy – Fatigue score; FACT-B HRQoL= Functional Assessment of Cancer Therapy – Breast Health-related quality of life; PA=physical activity; PFME=Pelvic Floor Muscle Exercises; PC=PeriCoach; ANCOVA=Analysis of Covariance; GDM=gestational diabetes mellitus; AUC=area under the curve; HOMA-IR= Homeostatic Model Assessment for Insulin Resistance; PSQI=Pittsburgh Sleep Quality Index; GAD-7= General Anxiety Disorder-7; IPAQ= International Physical Activity Questionnaire; CES-D= Center for Epidemiologic Studies Depression Scale
